# Sequencing as a first-line methodology for cystic fibrosis carrier screening

**DOI:** 10.1038/s41436-019-0525-y

**Published:** 2019-04-30

**Authors:** Kyle A. Beauchamp, Katherine A. Johansen Taber, Peter V. Grauman, Lindsay Spurka, Jeraldine Lim-Harashima, Ashley Svenson, James D. Goldberg, Dale Muzzey

**Affiliations:** 1 Myriad Women’s Health (formerly Counsyl), South San Francisco, CA USA; 2 Myriad Genetics, Salt Lake City, UT USA; 3 Present Address: Guardant Health, Redwood City, CA USA; 4 Present Address: Invitae, San Francisco, CA USA

**Keywords:** cystic fibrosis, carrier screening, sequencing

## Abstract

**Purpose:**

Medical society guidelines recommend offering genotyping-based cystic fibrosis (CF) carrier screening to pregnant women or women considering pregnancy. We assessed the performance of sequencing-based CF screening relative to genotyping, in terms of analytical validity, clinical validity, clinical impact, and clinical utility.

**Methods:**

Analytical validity was assessed using orthogonal confirmation and reference samples. Clinical validity was evaluated using the CFTR2 database. Clinical impact was assessed using ~100,000 screened patients. Three screening strategies were compared: genotyping 23 guideline-recommended variants (“CF23”), sequencing all coding bases in *CFTR* (“NGS”), and sequencing with large copy-number variant (CNV) identification (“NGS + CNV”). Clinical utility was determined via self-reported actions of at-risk couples (ARCs).

**Results:**

Analytical accuracy of NGS + CNV was 100% for SNVs, indels, and CNVs; interpretive clinical specificity relative to CFTR2 was 99.5%. NGS + CNV detected 58 ARCs, 18 of whom would have gone undetected with CF23 alone. Most ARCs (89% screened preconceptionally, 56% prenatally) altered pregnancy management, and no significant differences were observed between ARCs with or without at least one non-CF23 variant.

**Conclusion:**

Modern NGS and variant interpretation enable accurate sequencing-based CF screening. Limiting screening to 23 variants does not improve analytical validity, clinical validity, or clinical utility, but does fail to detect approximately 30% (18/58) of ARCs.

## INTRODUCTION

Cystic fibrosis (CF) is a serious hereditary condition affecting nearly 35,000 individuals in the United States.^1^ CF patients have increased morbidity and early mortality, primarily via progressive loss of lung function and infection. At the molecular level, CF is an autosomal recessive (AR) condition caused by two nonfunctional (or missing) copies of the *CFTR* gene, which encodes the cystic fibrosis transmembrane conductance regulator protein that regulates fluid transport and mucus accumulation in epithelial tissue. Though new CF treatments have recently been approved, challenges remain, such as extending the spectrum of treatable mutations and decreasing the price of treatment.^2,3^

As the most common life-threatening AR condition in non-Hispanic whites,^4^ CF is the subject of well-established carrier screening guidelines that predate the completion of the Human Genome Project. In the mid-1990s, the National Institutes of Health began work to develop screening guidelines.^5^ In 2001, 25 pathogenic variants were recommended by the American College of Medical Genetics and Genomics (ACMG) for population screening based on their frequency in the general population;^6^ a 2004 ACMG revision trimmed this list to focus on 23 common variants with confirmed pathogenicity.^7^ Recent American College of Obstetricians and Gynecologists (ACOG) guideline updates have reaffirmed the primacy of these 23 variants.^4^

Major scientific changes occurred in parallel with the development of CF screening guidelines. In 2003, the Human Genome Project reached completion.^8^ Soon thereafter, next-generation sequencing (NGS) technologies allowed low-cost interrogation of entire genomes, exomes, or gene panels.^9^ These technologies are increasingly used to facilitate diagnosis and screening of heritable cancer and other genetic disorders.^10,11^

Here we present four lines of evidence to evaluate the efficacy of NGS-based CF screening. First, analytical validation of an NGS-based carrier screen for CF demonstrates that modern NGS technology routinely achieves high sensitivity and specificity for identifying variants. Second, standardized variant interpretation processes—once highly heterogeneous—now enable laboratories to accurately perform real-time interpretation of variants observed in clinical sequencing;^12^ the accuracy of these processes (i.e., clinical validity) can be evaluated using actively curated community databases (e.g., CFTR2).^13^ Third, we show that CF23-only screening has negative clinical consequences, failing to detect approximately 30% of affected pregnancies—deficits that are disparate among US ethnicities. Finally, we show that a majority of CF at-risk couples (ARCs) identified via NGS of the entire *CFTR* coding region use knowledge of their CF risk status to alter reproductive decisions and pregnancy management, and that actions are not significantly different among ARCs carrying CF23 variants versus those carrying non-CF23 variants.

## MATERIALS AND METHODS

### Institutional review board

This study was reviewed and approved as exempt by Western Institutional Review Board (WIRB). Patient data were de-identified prior to analysis. Patients provided informed consent for testing and anonymized research.

### At-risk couples

In this work, we define at-risk couples (ARCs), also known as carrier couples, as couples for whom both partners are CF carriers. It is important to note that a couple not identified as an ARC still retains some level of residual risk after a negative screening result, so the ARC terminology should not be viewed as implying a binary assessment of risk status.

### Test and patient cohort

The study investigated anonymized results from a total cohort of up to 115,571 eligible patients tested with the NGS-based Foresight carrier screen (Myriad Women’s Health [formerly Counsyl], South San Francisco, CA), tested between 19 July 2017 and 14 May 2018. Patients were included in the study only if they received screening for CF; though they may also have received screening for additional expanded carrier screening (ECS) conditions, those results were not analyzed herein. Two analyses involved subsets of the total cohort. First, we examined all couples for whom both the male and female partners were tested and who received results as a couple report. This analysis identified 13,080 couples whose risk (i.e., ARC status) could be assessed. Second, for unbiased modeling of disease incidence, we examined the cohort of patients whose indication for testing on the requisition form was “routine carrier screening.” This cohort of 92,655 patients excludes patients with known family history and/or infertility, allowing a more accurate estimate of general population rates.^14,15^ Certain analyses also involved patients’ self-reported ethnicities; aggregate patient count by ethnicity is given in Table S1.

### NGS testing

Single-nucleotide variants (SNVs), short insertions and deletions (indels), and copy-number variants (CNVs, e.g., exon-level deletions and duplications) in the *CFTR* gene were identified via a previously described customized NGS-based ECS that uses hybridization capture to enrich for genes like *CFTR* and a bioinformatics pipeline to identify variants.^16^ SNVs and indels were called using GATK 1.6, Freebayes, and custom bioinformatics software. CNVs were called using previously described custom software that leverages read-depth information.^16,17^

### Analytical validation: general

Positive samples from 33 anonymized patients and 88 cell lines (from 1000 Genomes samples) were aggregated for orthogonal validation: variants were confirmed either internally (e.g., with Sanger sequencing for SNVs and indels, or with multiplex ligation-dependent probe amplification [MLPA] for CNVs), or via other published variant data, (e.g., 1000 Genomes data). A complete analytical validation of this ECS panel was recently published and contains several *CFTR* variants;^16^ here we re-examined analytical validity specifically in *CFTR* and augmented the previous validation samples with additional ones. Binomial parameter confidence intervals were estimated using the Jeffreys prior.

### Analytical validation: MLPA

To perform orthogonal validation of CNV-positive samples, MLPA was performed on 33 samples according to manufacturer’s protocol (MRC Holland, probemix P091-D2 *CFTR* protocol issued 13 March 2018 and MLPA General Protocol issued on 23 March 2018).

### Clinical validity

*CFTR* variants were interpreted using an ACMG-compliant process,^12^ with classification criteria specifically tailored for CF. Recent versions of these criteria utilize the actively curated CFTR2 database as well as other databases.^13,18^

To assess clinical validity, Foresight *CFTR* variant interpretations were compared with the CFTR2 public database (accessed August 2018) of common disease-causing *CFTR* variants.^13^ First, all variants observed in the patient cohort and classified as pathogenic by Foresight were compiled. Then, the CFTR2 search feature was used to find a matching variant in*CFTR*. All variants, with their CFTR2 links, are provided in Table S3. The specificity of Foresight pathogenic classifications was estimated assuming that CFTR2 is a truth set. Variants were considered a match between the two databases if at least one variant name (technical or common name) matched. Our approach to evaluate clinical validity focuses on the variant classification specificity of NGS + CNV because this was a published area of concern;^19^ though not explicitly calculated here, clinical sensitivity is guaranteed to be higher than CF23 because the CF23 variants are included on the NGS + CNV panel.

### Clinical impact

Clinical impact was assessed in two ways: (1) counting ARCs among the Foresight patient cohort and (2) modeling population-level disease incidence using the modeled fetal disease risk approach described previously.^14,15^ Modeling assumed intraethnicity coupling, consistent with our previous incidence-modeling studies.^14–16^ This modeling approach yields incidence estimates concordant with those measured without modeling; see, e.g., Table S8 in ref. ^20^ with the caveat that this previous study excluded variable penetrance CF.

### Clinical utility

To measure the clinical utility of CF carrier screening for pathogenic variants beyond those recommended by guidelines, previously described methods were used for survey development, cohort determination, survey fielding, and data collection.^21^ This cohort included 37 CF ARCs screened on one of two NGS-based carrier screening platforms at Counsyl/Myriad Women’s Health, one that sequenced all *CFTR* exons without CNV calling (now a deprecated platform), or one that sequenced all*CFTR* exons with CNV analysis (the current platform). Exact counts of survey respondents who were screened with each platform are unavailable because this information was purposefully excluded during cohort anonymization.

To quantify actions planned or pursued as a result of positive CF carrier screening results, data analyses were performed on ARCs that had a high risk of current or future pregnancies being affected with CF (i.e., both the mother and father were carriers of pathogenic variants in *CFTR*). Couples that were ARCs for conditions in addition to CF were excluded to isolate the impact of CF ARC status. Statistical significance between proportions was determined using Fisher’s exact test; a result was considered significant when *P* < 0.05.

## RESULTS

### Analytical validity

**Fig. 1:**
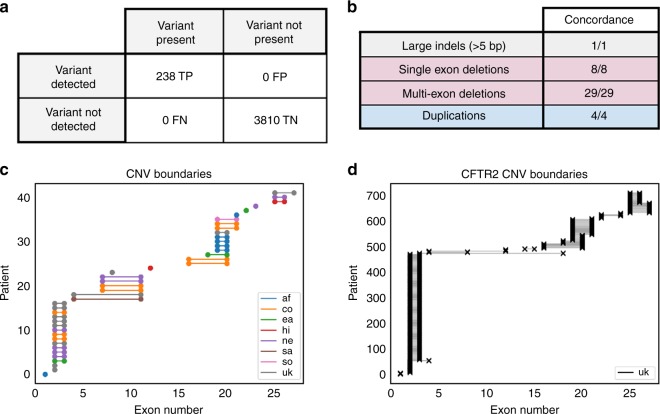
Analytical Validity. **a** Analytical sensitivity and specificity for single-nucleotide polymorphism (SNP) and small indel variants in *CFTR*, as assessed on 4048 allele calls among 88 samples (70 of which were positive). As assessed per variant, observed sensitivity and specificity were both 100%, with 95% confidence lower bounds of 99.0% and 99.9%, respectively. As assessed per patient, observed sensitivity and specificity were both 100%, with 95% confidence lower bounds 96.5% and 97.2%, respectively. **b** Analytical concordance (positive samples) for other variant classes in*CFTR*. **c**–**d** Exonic position distribution of *CFTR* copy-number variants (CNVs) observed in screening (**c**; Foresight) and diagnostic (**d**; CFTR2) settings. Start and end positions were estimated from Human Genome Variation Society (HGVS) and/or common names using nonlegacy exon numbering. In **c**, the*y*-axis represents distinct individuals in the cohort, such that each patient corresponds to a distinct horizontal line. *af* African or African-American,*co* Mixed or Other Caucasian, *ea* East Asian,*FN* false negative,*FP* false positive,*hi* Hispanic, *ne* Northern European, *sa* South Asian, so Southern European, *TN* true negative,*TP* true positive,*uk* Unknown.

We sought to evaluate whether an NGS-based screen for CF carriers achieved an acceptable level of analytical sensitivity and specificity. A previous analytical validation study of the 235-gene Foresight ECS panel demonstrated >99.99% sensitivity and specificity for SNV/indel calling (assessed on >200,000 calls) and perfect concordance for rarer variant classes (large indels and CNVs).^16^ We revisited that analysis in two ways. First, we restricted the SNV/indel performance to include only calls within*CFTR*, which revealed no false positives or false negatives for >2000 exonic variant calls in 88 samples (metrics and confidence intervals in Fig. 1a and its legend). Second, we augmented the previous data set with 33 additional CNVs that were observed in 33 patients (Table S2) and for which sufficient DNA remained for reanalysis; orthogonal confirmation via MLPA verified the NGS results for all CNVs (Fig. 1b). As a simultaneous verification of both the analytical and clinical validity of CNV detection in*CFTR*, we observed that the positional distribution of CNVs reported in NGS-based carrier screening was similar to that found in diagnostic sequencing of CF patients (Fig. 1c, d). Finally, we additionally used computer simulations, based on empirically observed depth variability, to show that CNV sensitivity is expected to be high and robust to laboratory variability (see Figures S1–S3 and Supporting Information).

Taken together, the perfect concordance data for SNVs, indels, and CNVs demonstrated that NGS + CNV is an analytically valid manner of detecting variants in *CFTR*.

### Clinical validity

We next investigated the clinical validity of the NGS-based CF screen by evaluating the specificity of the variant interpretation process. As noted in “Materials and methods,” clinical sensitivity of NGS + CNV is expected to exceed that of CF23; therefore, the clinical validity assessment focused on variant classification specificity because it has been directly questioned and was as-yet unestablished.^19^ We leveraged the CFTR2 database as a truth set because it is based on over 89,000 diagnosed CF patients with known sequencing results and augments patient-level clinical data with in vitro data; accordingly, it is the gold standard of CFTR variant interpretations (pathogenic, uncertain significance [VUS], or variable). We excluded the common NM_000492.3(CFTR):c.1210–12T[5] variant because it is pathogenic only in the presence of select other variants. We also did not include variants considered VUS or benign (by Foresight), as these are not reported to carrier screening patients and, therefore, would not compromise NGS specificity.^22^ There were 3965 observed pathogenic alleles in the cohort (Table 1, top; note that, e.g., 15 patients with the same variant counted as 15 alleles), and 98% (3884/3965) were also present in the CFTR2 database. Among alleles in both databases, 99.5% (3865/3884) were determined to be pathogenic by CFTR2, suggesting that NGS-based screening for CF has high clinical specificity. The only mismatch was NM_000492.3(CFTR):c.2657+2_2657+3insA (“2789 + 2insA”), considered a VUS by CFTR2 but pathogenic by Foresight; curation details for this variant are provided in the Supplementary Information. The 3965 reported pathogenic alleles were clustered among 213 unique variants (Table 1, bottom); 45 variants were classified as pathogenic in Foresight but excluded from CFTR2, which focuses on common CF variants. We calculated specificity—99.5% from above—using allele frequencies rather than the number of unique alleles because the latter artificially inflates clinical impact: the unique alleles omitted from CFTR2 tended to be rare, often present in only a single patient in the cohort (see Supplementary Information for an analysis at the unique variant level).

**Table 1:** Clinical specificity is assessed by concordance to the CFTR2 database

		CFTR2		
		Pathogenic	VUS	Missing
Foresight	# of pathogenic alleles	3865	19	81
	# of unique pathogenic variants	167	1	45

*VUS* variant of uncertain significance.

As an even more stringent test of the incremental impact on clinical validity of CF carrier screening via NGS rather than via genotyping, we assessed results after excluding CF23 variants. Among alleles observed in both databases, 97.5% (735/754) agreement was observed with CFTR2. Finally, we asked whether alleles marked as having variable penetrance by CFTR2 were also annotated by Foresight as having variable penetrance. Among reported alleles, 99.5% (560/563) were found to have variable penetrance via both CFTR2 and Foresight.

In summary, NGS-based CF screening reports variants with high specificity (99.5%; confidence bounds [99.3%, 99.7%]); of 115,571 screened patients, only 19 reported pathogenic calls (19 patients) were discordant with CFTR2.

### Clinical impact

To assess the clinical impact of NGS-based CF carrier screening, we used the following hypothetical: “If Foresight patients had received more limited CF screening, how many missed cases would result?” For this analysis, we compared three levels of CF screening in both the ARC cohort and in a modeled US population: CF23 genotyping (“CF23”), CF sequencing without CNV analysis (“NGS”), and CF sequencing with CNV analysis (“NGS + CNV”).

**Fig. 2:**
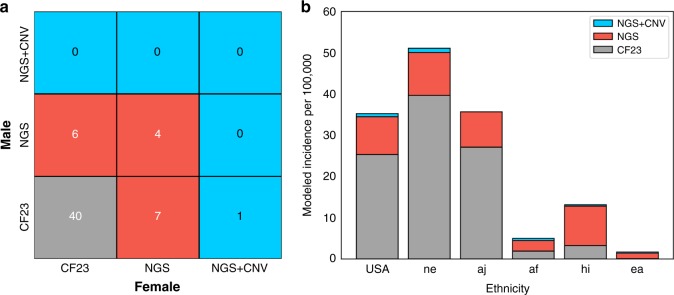
Clinical Impact. **a** At-risk couples (ARCs) were grouped by the three *CFTR* variant classes and counted by the minimal screening methodology required to detect the male and female carried variants. **b** Cystic fibrosis (CF) incidence was modeled separately for several relevant ethnicities and collectively for a US-weighted population. *af* African or African-American, *aj* Ashkenazi Jewish, *CNV* copy-number variant, *ea* East Asian, *hi* Hispanic, *ne* Northern European, *NGS* next-generation sequencing.

Among 13,080 couples screened, 58 were CF ARCs (Fig. 2a), and 40 of those would have been detected by CF23-only screening. The risk faced by the remaining 18 couples would have been missed, resulting in approximately 4–5 additional CF cases. Notably, CNV analysis was required to detect one of these 18 ARCs. Stated another way, the usage of CF23 genotyping fails to detect 31% (18/58) of ARCs. These 18 couples were diverse in ethnicity, with the most commonly observed ethnicities including Mixed or Other Caucasian, Northern European, South Asian, and Hispanic; none of the partners in these 18 couples were Ashkenazi Jewish. In many clinical settings, positive carrier status of a first partner screened with CF23 will lead to full CF sequencing for the subsequent partner. This approach, which, to our knowledge, is not recommended by existing guidelines, would recover detection for 6 of the 18 ARCs, yet 12 would still have gone undetected if the female partner received CF23 screening.

Next, we modeled CF incidence and the efficacy of different screening approaches in a US population (Fig. 2b), as well as in several ethnicities. As expected, incidence varied by ethnicity, with Ashkenazi Jewish and Northern European persons showing the highest rates. Likewise, the efficacy of CF23-only screening varied by ethnicity. In Ashkenazi Jewish persons, CF23 accounted for 76% of affected pregnancies. In the US population, this number fell to 72%. Finally, in Hispanic persons—for whom the absolute risk of CF is relatively high (modeled herein as 13 affected pregnancies per 100,000 pregnancies)—CF23 screening identified only 25% of affected pregnancies. Additionally, the relative gain of adding CNV calling to NGS was impactful. In the modeled US population, the addition of CNVs led to a 2% gain in detection (i.e., incidence), comparable with the 1.8% (58/57) gain observed directly in ARCs. The detection gain driven by CNVs was also ethnicity-specific: eight pathogenic CNVs were detected among 16,087 Northern European persons while no pathogenic CNVs were observed among the 5703 Ashkenazi Jewish persons screened. Observed pathogenic CNVs were primarily deletions (30/33 alleles and 17/18 variants) and were observed across several exons (see Fig. 1 and Table S2).

### Clinical utility of CF screening

**Fig. 3:**
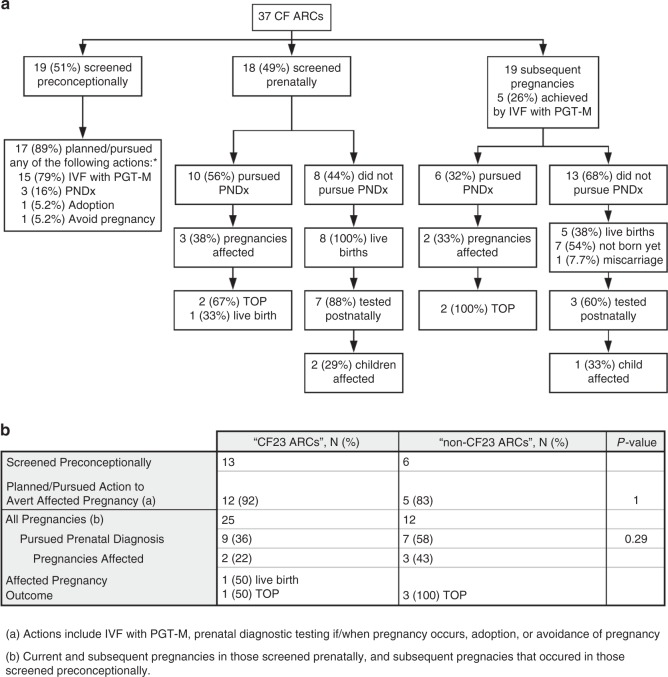
Clinical Utility. Actions planned or pursued by cystic fibrosis (CF) at-risk couples (ARCs) as a result of knowing their CF risk status **a**, and comparison of actions planned or pursued by couples carrying variants that are included on the CF23 panel versus outside of the panel **b**. *IVF* in vitro fertilization, *PGT-M* preimplantation genetic testing for monogenic conditions, *PNDx* prenatal diagnostic testing, *TOP* termination of pregnancy. Asterisk (*) indicates ARCs could choose more than one action, so percentages sum to more than 100%.

The clinical utility of CF carrier screening was measured by determining how many ARCs identified by NGS-based screening modified reproductive decisions and pregnancy management based on knowledge of their risk status. Results are summarized in Fig. 3a. Among ARCs not pregnant when they received ECS results, 89% planned or pursued actions to reduce the risk of having a CF-affected pregnancy, including in vitro fertilization (IVF) with preimplantation genetic testing for monogenic conditions (“IVF with PGT-M”; 79%), prenatal diagnostic testing if/when they became pregnant (16%), adoption (5.2%), and avoidance of pregnancy (5.2%). Among those who were pregnant when they received ECS results, 56% pursued prenatal diagnostic testing (Fig. 3a); three pregnancies were found to be affected, two of which were discontinued and one of which resulted in a live birth. More than one-quarter of pregnancies conceived subsequent to receiving carrier screening results were achieved by IVF with PGT-M (26%). Six (32%) pregnancies underwent prenatal diagnostic testing (Fig. 3a). Two were found to be affected; both pregnancies were discontinued.

Among ARCs who declined prenatal diagnostic testing and whose pregnancies resulted in live births, 88% screened prenatally had undertaken diagnostic testing after birth (Fig. 3a). In subsequent pregnancies (for those screened prenatally, each pregnancy after the one during which they were screened; for those screened preconceptionally, every pregnancy that occurred after they were screened), 60% had undertaken diagnostic testing after birth (Fig. 3a).

We also compared the actions taken by ARCs in which both members carry CF23 variants (“CF23 ARCs”) versus those in which at least one member carries a variant outside of CF23 (“non-CF23 ARCs”) to determine whether actions planned or pursued were different between the two groups. The proportion of preconceptionally screened non-CF23 ARCs who took action to avert an affected pregnancy (83%) was not significantly different from that of CF23 ARCs who took action (92%) (Fig. 3b). Among all pregnancies, 58% of non-CF23 ARCs pursued prenatal diagnostic testing compared with 36% of CF23 ARCs, proportions that also were not significantly different (Fig. 3b).

## DISCUSSION

Here we used data from >100,000 anonymized patients to evaluate the analytical and clinical performance of sequencing-based carrier screening for CF. As assessed using orthogonal confirmation and simulation, analytical accuracy is high (100% in the current study). Clinical specificity is also high (99.5% in the current study), as measured by variant interpretation concordance to the CFTR2 database. We have shown that restricted, CF23-only screening fails to detect a substantial number of ARCs, particularly for non–Ashkenazi Jewish ethnicities. Finally, our results confirm the clinical utility of NGS-based CF carrier screening in preconception, prenatal, postnatal, and subsequent-pregnancy settings. We observed that a substantial number of CF ARCs used knowledge of their CF risk status to undertake actions that reduced the incidence of live births affected with CF.

### Does NGS lead to more false positives?

One argument made against using sequencing for CF carrier screening is that it introduces a large number of false positives that would be avoided via CF23 screening.^19^ This argument often has two variations. First, one might argue that sequencing technology has a nonzero error rate at any given site and that the probability of finding a false positive increases as one searches for more variants across the many sites of a larger genomic territory (a multiple hypothesis testing argument). Second, one might argue that clinical false positives (i.e., from misinterpreted variants) limit specificity.

With current and appropriately validated NGS approaches and classification workflows, neither of these two arguments holds true. A previous analytical validation study of the ECS panel used herein showed that analytical errors are rare: one false positive call was made while 212,139 true negative calls were made. Regarding false positives that could arise from broadened genomic purview, it is important to note that carrier screening generally reports only pathogenic variants (our laboratory follows the joint recommendation stating that VUS should not be reported to carrier screening patients),^22^ and in the present study, despite full-exon sequencing of *CFTR*, only a few hundred variants were deemed pathogenic via the rigorous classification process. Among those variants that were reported, specificity in variant interpretation was shown here to be high, as measured by concordance to CFTR2.

### Should we screen rare CF variants?

A previous commentary noted challenges in obtaining accurate allele frequencies and phenotype correlations for rare variants,^19^ suggesting that unreliable data would lead to selection of arbitrary variants for screening.

The concern about rare CF alleles populating the list of screened variants does not apply to NGS-based CF screening. First, NGS-based screening has no list of screened variants; instead, the region of interest (*CFTR* exons and limited, functional portions of adjacent introns) is sequenced, and variants in that region classified as pathogenic based on current evidence are reported. Second, the risk of rare variants lowering specificity in NGS-based screening is mitigated due to the variant classification process: because an allele’s frequency influences the availability of case–control statistics used during the variant interpretation process, rare variants that have insufficient clinical data are classified as VUS and go unreported.^12,23^ Using a strict allele frequency cutoff to exclude rare variants from screening may instead lead to reduced detection of ARCs (as some variants with low frequency nevertheless have enough experimental and clinical evidence to be classified as pathogenic). Thus, one could expect reduced clinical sensitivity, but likely no impact on clinical specificity.

### Can we avoid a CF “arms race”?

Some have warned against a so-called arms race in CF screening, where laboratories compete to screen the most variants.^19^ We agree that such a scenario is unadvisable and propose the following to avoid it. First, the number of variants should not be used as a comparator; instead, the focus should always be on sensitivity and specificity (either analytical or clinical) of known pathogenic variants. Second, laboratories should demonstrate adherence to guideline-based classification practices, which place a natural guard against an unsubstantiated uptick in the number of variants: due to the stringency of variant interpretation (guidelines recommend even stricter criteria for a pathogenic classification when screening an unaffected population as in carrier screening),^12^ reports from the present cohort of >100,000 patients included only 213 unique pathogenic variants.

### Do CF ARCs modify reproductive decisions and pregnancy management based on their CF risk status?

Current medical society guidelines state that routine NGS-based CF carrier screening is not appropriate, and, consistent with this point of view, several payer medical policies support sequencing only in special situations.^24,25^ The clinical utility of CF carrier screening and ECS has been demonstrated previously;^21,26^ here we add to the clinical utility evidence by showing that CF ARCs identified by NGS-based screening modified their reproductive decisions based on knowledge of their CF risk status.

Two key results demonstrate that NGS-based screening for CF risk status has clinical utility. First, the vast majority of CF ARCs planned or pursued actions to reduce the risk of having a CF-affected birth, implying a reduced incidence of CF. Second, knowing CF carrier status facilitated targeted postnatal diagnostic testing among the majority of those who chose not to undergo prenatal diagnostic testing, establishing a CF diagnosis in three children and ruling out a diagnosis in seven children.

We also show that ARCs take action to a comparable extent whether they carry a CF23 variant or a variant outside of CF23. Of CF ARCs included in the survey cohort, 31% (18/58) would have gone undetected by CF23-only screening. In this group, three affected pregnancies could have gone undetected, eliminating the chance for ARCs to make informed choices to manage their affected pregnancies.

### Limitations

The following are limitations of the present study. The assessment of clinical validity relied on three assumptions. First, we evaluated clinical validity only through the lens of concordance in variant interpretations. This assumption is likely reasonable because variant interpretation encompasses much of the concern about NGS-based carrier screening and because other aspects of clinical validity (e.g., the link between the gene and a disease phenotype) are well established for CF. Second, the analysis relies on the accuracy of the CFTR2 database. However, comparison with other public databases has revealed similar estimates of clinical accuracy, suggesting that this approach is reasonably robust.^15^ Third, comparisons to databases (e.g., CFTR2) are not totally independent, as the databases themselves are used during variant interpretation as a source of CF cases, associated phenotypes, and functional studies. However, this challenge is unavoidable, as proper patient care requires that variant interpretations use the most accurate data available, which in many cases includes patient data from databases like CFTR2.

Regarding the evaluation of clinical utility, the present study has two primary limitations. First, survey results relied on patient recall, which can be inaccurate. Second, there is a possibility of response bias, where participants who took action based on their CF carrier results were more likely to have completed the survey than those who did not take action. Nevertheless, the rate of reproductive intervention reported here is similar to those reported across several other studies and cohorts,^21,27,28^ suggesting that our results are generalizable.

### Conclusion

Collectively, the analytical validity, clinical validity, clinical impact, and clinical utility data presented here support NGS-based CF carrier screening for patients of reproductive age who are planning to conceive or are already pregnant. Broader support for such screening could detect more ARCs than existing CF23-only guidelines.

## Supplementary information

appendix_s4

Supplementary Information

Supplemental Table S3
