# Correction: Sequencing as a first-line methodology for cystic fibrosis carrier screening

**DOI:** 10.1038/s41436-019-0543-9

**Published:** 2019-05-15

**Authors:** Kyle A. Beauchamp, Katherine A. Johansen Taber, Peter V. Grauman, Lindsay Spurka, Jeraldine Lim-Harashima, Ashley Svenson, James D. Goldberg, Dale Muzzey

**Affiliations:** 1 Myriad Women’s Health (formerly Counsyl), South San Francisco, CA USA; 2 Myriad Genetics, Salt Lake City, UT USA; 3 Present Address: Guardant Health, Redwood City, CA USA; 4 Present Address: Invitae, San Francisco, CA USA

Correction to: *Genetics in Medicine;* 10.1038/s41436-019-0525-y, published online 30 April 2019

The original version of this Article contained an error in Fig. 3. Specifically, the result “3 (67%) TOP” should read “2 (67%) TOP.” This has now been corrected in both the PDF and HTML versions of the Article.

**Figure 1:**
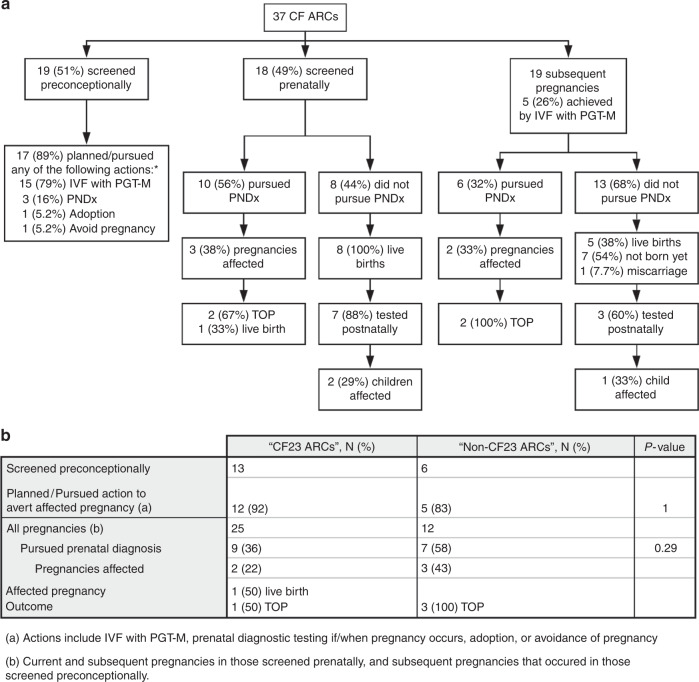
**Fig. 3**

