# Supplementary material for: Sequencing as a first-line methodology for cystic fibrosis carrier screening
Source: Genet Med. 2019 Apr 30;21(11):2569–76. doi: 10.1038/s41436-019-0525-y (PMC6831513; doi:10.1038/s41436-019-0525-y)
Supplement: Supplementary file 2 — Supplementary Information [file 41436_2019_525_MOESM2_ESM.docx]

# Sequencing as a first-line methodology for cystic fibrosis carrier: Supplementary Information

###

# Supplementary Information

### Analytical Validation: CNV Simulation Methods

Synthetic single-copy duplications and deletions were introduced by modifying the number of observed reads in a CNV-negative sample, as described previously [^1^](https://paperpile.com/c/nyOyUO/CaBnr). Deletions were introduced by subsampling reads to 50% of those originally observed; duplications were introduced by scaling reads to 150% of those originally observed. Thirty-five synthetic CNVs were introduced in *CFTR* for every exon and for every contiguous exon combination in the gene. CNV sensitivity for each exon combination was then calculated as the percentage of the synthetic CNVs that were correctly detected.

To assess the robustness of CNV sensitivity as a function of average NGS depth and the number of samples processed together in the same batch, the NGS dataset used for CNV simulations was modified before executing the above procedures. To simulate a sample that spuriously receives low depth, the depth was subsampled across the whole sample prior to CNV simulations. To simulate the impact on a single sample of a batch with many failed samples, various numbers of passing samples were used for background normalization.

#

# Analytical Validation: CNV Simulation Results

# In addition to demonstrating concordance and high specificity for all CNV-positive patients, we further characterized the sensitivity of CNV discovery. Because existing patient and reference samples do not completely cover the range of possible CNV variants, we assessed the performance of all possible single and multi-exon CNVs via simulation (Fig. S3a,b; see CNV Simulation Methods). Multi-exon CNVs were predicted to have near-100% sensitivity for all possible locations across the gene. Single-exon deletions (a) and duplications (b) also had high sensitivity (median of 100% and 86.2% for single-exon deletions and duplications, respectively), though some exons and duplications had somewhat reduced sensitivity. These trends were expected: duplications are harder to detect than deletions of equivalent size [^2^](https://paperpile.com/c/nyOyUO/VqtAQ), while certain exons may have sensitivity differences due to their size and sequencing efficiency. Importantly, the detection of CNVs was robust; sensitivity remained >90% even if a sample spuriously received only 50% of the intended sequencing data or if >50% of other samples—processed in parallel and leveraged for CNV discovery—had failed (Fig. S1).

### Curation Details for NM_000492.3(CFTR):c.2657+2_2657+3insA

NM_000492.3(CFTR):c.2657+2_2657+3insA is an intronic variant classified as likely pathogenic as assessed in the context of carrier screening. This variant has been observed in cases with cystic fibrosis and a pathogenic variant in trans (PMID: 16189704, 17283574, 23168765, 24586523, 15754262, 29055982) and in additional alleles of unspecified zygosity from cases with cystic fibrosis (PMID: 16963320, 15681482, 23974870, 26708955). A minigene splicing assay shows that this variant has a minimal effect on splicing (PMID: 25066652). Splicing prediction tools predict an effect on splicing and CADD predicts this variant is likely deleterious (phred score = 16.28). c.2657+2_2657+3insA has been observed in population frequency databases (ExAC NFE 0.003%, 0 homozygotes; GnomAD NFE 0.013%, 0 homozygotes). In summary, although the functional evidence shows only a minimal effect on splicing, the number of reported cases with cystic fibrosis compared to the low frequency of the variant in healthy controls suggest that this variant is likely to be disease-causing.
Unique Variant Clinical Validity Analysis

The clinical validity analysis in the main text focused on the number of alleles observed. Here we revisit that analysis in terms of number of unique variants, without accounting for the fact that some variants occur infrequently. There were 213 unique variants observed in our cohort. Of them, 79% (168 / 213) were present in CFTR2. Of those present in CFTR2, 99.4% (167 / 168) were found pathogenic by CFTR2. 7 variants were determined to have variable penetrance by CFTR2; 86% (6/7) were also annoted in Foresight as having variable penetrance.

Separately, we also examined the relative distribution of known, likely, and predicted pathogenic variants (i.e., the sub-categories of pathogenic variants). Among the pathogenic variants, we observed that 33% were known pathogenic, 27% were likely pathogenic, and 40% were predicted pathogenic.

As another stringent test, we examined the 9 variants found to be VUS by CFTR2, as assessed in the Aug. 31, 2018 CFTR2 statistics. Of these, Foresight would lead to the same clinical report for 8/9 variants; the one discordance was for NM_000492.3(CFTR):c.2657+2_2657+3insA, which Foresight interprets as likely pathogenic. Finally, two variants found to be VUS by CFTR2 were found to be likely benign by Foresight; this difference does not have clear clinical impact, as both VUS and benign variants are not reported in routine carrier screening. The full list of variants is tabulated below.

| **Foresight Name** | **Foresight Curation** | **CFTR2 Curation** | **CFTR2 URL** |
| --- | --- | --- | --- |
| NM_000492.3(CFTR):c.92G>T(R31L) | UNKNOWN | UNKNOWN | [https://www.cftr2.org/mutation/scientific/R31L](https://www.cftr2.org/mutation/scientific/R31L) |
| NM_000492.3(CFTR):c.164+28A>G | LIKELY_BENIGN | UNKNOWN | [https://www.cftr2.org/mutation/scientific/296%252B28A-%253EG/](https://www.cftr2.org/mutation/scientific/296%252B28A-%253EG/) |
| NM_000492.3(CFTR):c.601G>A(V201M) | UNKNOWN | UNKNOWN | [https://www.cftr2.org/mutation/scientific/V201M/](https://www.cftr2.org/mutation/scientific/V201M/) |
| NM_000492.3(CFTR):c.958T>G(L320V) | UNKNOWN | UNKNOWN | [https://www.cftr2.org/mutation/scientific/L320V/](https://www.cftr2.org/mutation/scientific/L320V/) |
| NM_000492.3(CFTR):c.1046C>T(A349V) | UNKNOWN | UNKNOWN | [https://www.cftr2.org/mutation/scientific/A349V/](https://www.cftr2.org/mutation/scientific/A349V/) |
| NM_000492.3(CFTR):c.2620-26A>G | LIKELY_BENIGN | UNKNOWN | [https://www.cftr2.org/mutation/scientific/2752-26A-%253EG/](https://www.cftr2.org/mutation/scientific/2752-26A-%253EG/) |
| NM_000492.3(CFTR):c.2657+2_2657+3insA | LIKELY_DELETERIOUS | UNKNOWN | [https://www.cftr2.org/mutation/scientific/2789%252B2insA/](https://www.cftr2.org/mutation/scientific/2789%252B2insA/) |
| NM_000492.3(CFTR):c.2735C>T(S912L) | UNKNOWN | UNKNOWN | [https://www.cftr2.org/mutation/scientific/S912L/](https://www.cftr2.org/mutation/scientific/S912L/) |
| NM_000492.3(CFTR):c.3041A>G(Y1014C) | UNKNOWN | UNKNOWN | [https://www.cftr2.org/mutation/scientific/Y1014C/](https://www.cftr2.org/mutation/scientific/Y1014C/) |

#

**Table S1.** Patient count by ethnicity for the “routine carrier screening” cohort (e.g., excluding patients with family history and/or infertility).

| **ETHNICITY** | **COUNT** | **Full Ethnicity** |
| --- | --- | --- |
| **af** | 7397 | African or African-American |
| **aj** | 5703 | Ashkenazi Jewish |
| **co** | 22725 | Mixed or Other Caucasian |
| **cj** | 298 | French Canadian or Cajun |
| **ea** | 4549 | East Asian |
| **fi** | 20 | Finnish |
| **hi** | 7861 | Hispanic |
| **me** | 1257 | Middle Eastern |
| **na** | 170 | Native American |
| **ne** | 16087 | Northern European |
| **pi** | 146 | Pacific Islander |
| **sa** | 3439 | South Asian |
| **se** | 1469 | Southeast Asian |
| **so** | 1726 | Southern European |
| **uk** | 19808 | Unknown |
| **ALL** | 92655 |  |

**Table S2: Observed pathogenic CNVs observed in patient cohort that underwent MLPA confirmation. All CNVs were confirmed with MLPA.**

| **Variant** | **Counts** |
| --- | --- |
| **NM_000492.3(CFTR): Exon 19-20 del(aka c.(2988+206_2989-63)_(3367+101_3368-80)del)** | 5 |
| **NM_000492.3(CFTR): Exon 2-3 del(aka c.(53+394_54-6142)_(273+10330_274-221)del)** | 5 |
| **NM_000492.3(CFTR): Exon 7-11 dup(aka c.(743+81_744-297)_(1584+454_1585-9187)dup)** | 3 |
| **NM_000492.3(CFTR): Exon 2-3 del(aka c.(54-5833_54-255)_(273+10330_274-221)del)** | 3 |
| **NM_000492.3(CFTR) : Exon 2 del(aka c.(54-5833_54-255)_(164+124_165-265)del)** | 2 |
| **NM_000492.3(CFTR): Exon 19-21 del(aka c.(2988+206_2989-63)_(3468+210_3469-223)del)** | 2 |
| **NM_000492.3(CFTR):Exon 25-26 del(aka c.(3963+77_3964-281)_(4242+152_4243-224)del)** | 2 |
| **NM_000492.3(CFTR): Exon 4-11 del(aka c.(273+10330_274-221)_(1584+454_1585-9187)del)** | 1 |
| **NM_000492.3(CFTR): Exon 16-20 del(aka c.(2619+369_2620-306)_(3367+101_3368-80)del)** | 1 |
| **NM_000492.3(CFTR): Exon 21 del(aka c.(3367+101_3368-80)_(3468+210_3469-223)del)** | 1 |
| **NM_000492.3(CFTR): Exon 2-3 del(aka c.(54-6102_54-5873)_(273+334_273+10183)del)** | 1 |
| **NM_000492.3(CFTR): Exon 2-3 del(aka c.(54-5833_54-255)_(273+334_273+10183)del)** | 1 |
| **NM_000492.3(CFTR): Exon 8 del(aka c.(870-1053_870-126)_(1116+186_1117-409)del)** | 1 |
| **NM_000492.3(CFTR): Exon 23 del(aka c.(3717+250_3718-2695)_(3873+162_3874-4582)del)** | 1 |
| **NM_000492.3(CFTR): Exon 2-3 del(aka c.(54-6102_54-5873)_(273+10330_274-221)del)** | 1 |
| **NM_000492.3(CFTR): Exon 1 del(aka c.(-992_-951)_(53+394_54-6142)del)** | 1 |
| **NM_000492.3(CFTR): Exon 12 del(aka c.(1585-9147_1585-120)_(1679+214_1680-1113)del)** | 1 |
| **NM_000492.3(CFTR): Exon 22 del(aka c.(3468+210_3469-223)_(3717+250_3718-2695)del)** | 1 |

**Figure S1.** The robustness of single-exon deletion sensitivity was assessed by synthetically reducing the number of samples per lane and per-sample sequencing depth.


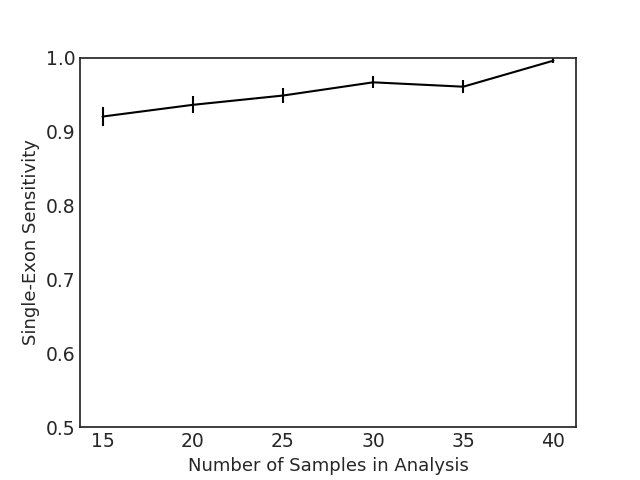

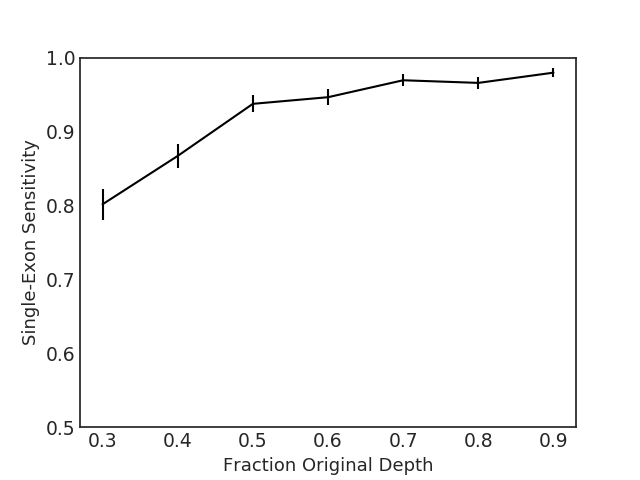
**Figure S2**. The CNV caller log odds ratio (LOD) for whether a sample is wildtype was calculated for CNV-positive and CNV-negative samples to show that positive calls are well-separated from negative calls.


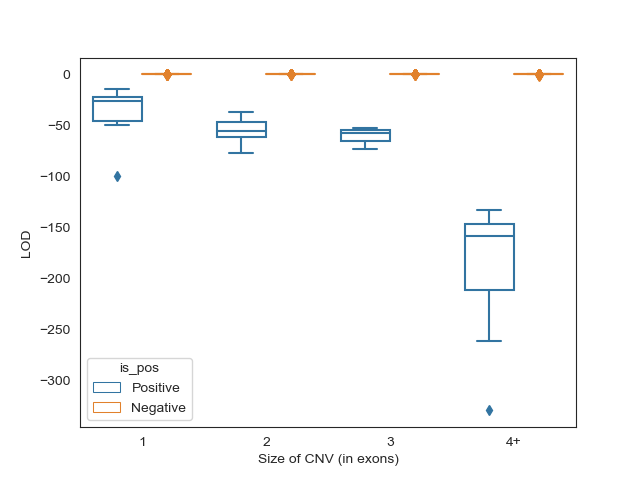


**Figure S3.** (a). Simulated sensitivity for all possible exon-level *CFTR* deletions. (b). Simulated sensitivity for all possible exon-level *CFTR* duplications.


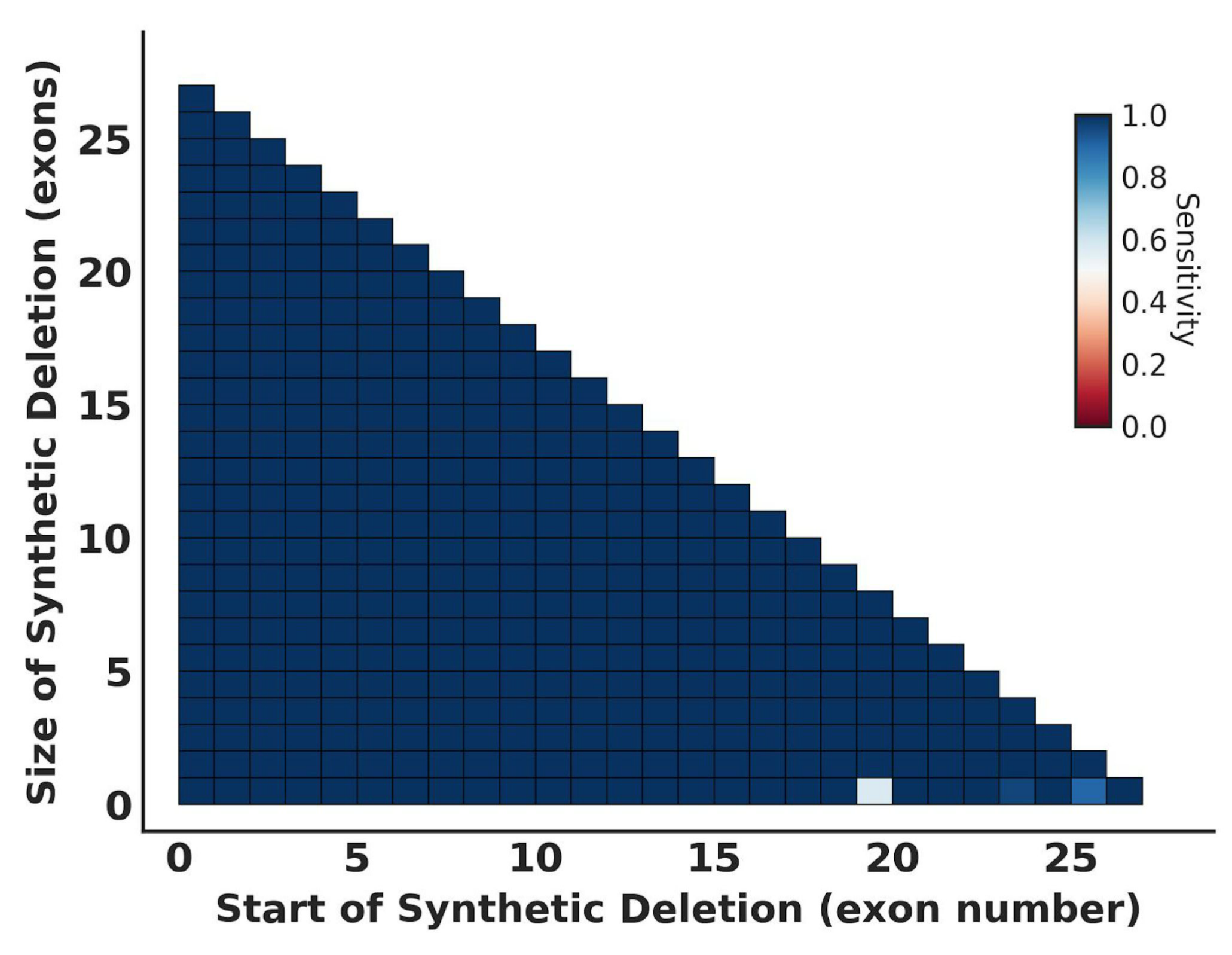

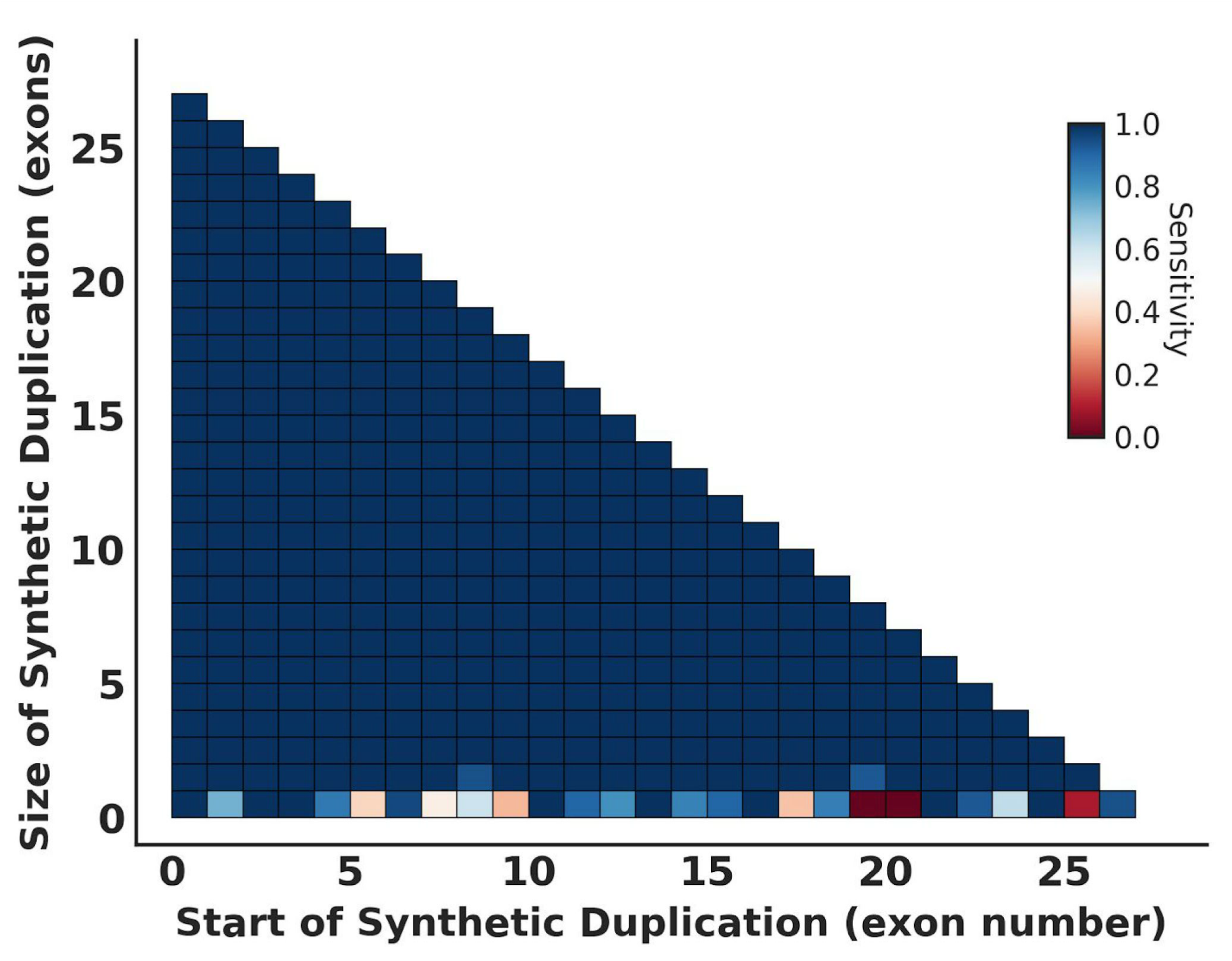


**Table S3.** All pathogenic variants observed in this cohort are listed, as well as the CFTR2-reported pathogenicity. Links to the CFTR2 pages are provided.

(Note: table only viewable in SI Excel file, as it is too large to copy in SI text)

Appendix S4. The ARC survey used for clinical utility assessment is included as a Word document.

# References

1. [Hogan, G. J. *et al.* Validation of an Expanded Carrier Screen that Optimizes Sensitivity via Full-Exon Sequencing and Panel-wide Copy Number Variant Identification. *Clin. Chem.* (2018). doi:](http://paperpile.com/b/nyOyUO/CaBnr)[10.1373/clinchem.2018.286823](http://dx.doi.org/10.1373/clinchem.2018.286823)

2. [Oldridge, D. A., Banerjee, S., Setlur, S. R., Sboner, A. & Demichelis, F. Optimizing copy number variation analysis using genome-wide short sequence oligonucleotide arrays. *Nucleic Acids Res.* **38**, 3275–3286 (2010).](http://paperpile.com/b/nyOyUO/VqtAQ)
